# Massachusetts Veterinary Medical Association

**Published:** 1897-03

**Authors:** Henry S. Lewis

**Affiliations:** Secretary


### MASSACHUSETTS VETERINARY MEDICAL ASSOCIATION.

THE regular monthly meeting of this Association was held at 19 Boylston Place on Wednesday evening, December 23, 1896. The President, Dr. John M. Parker, called the meeting to order at 8.15 P.M. Members present were Drs. Cronan, Frothingham, Hamilton, Howard, Lee, Lewis, McLaughlin, Pierce, Rogers, Stickney, Williams, Winchester, and Winslow.

The minutes of previous meeting were adopted. Dr. Peters reported for the committee appointed at the November meeting that they had sent memorials to the United States Senators and each Congressman of Massachusetts urging the passage of the Veterinary Surgeons' U. S. Army Bill—a bill to fix the pay, allowances, tenure of office, and rank of veterinary surgeons of the U. S. Army.

Dr. Winchester moved that the report of the committee be accepted; carried.

Dr. W. D. Plaskett, of Clinton, and Dr. R. E. Dyer, of South Boston, were elected members.

Dr. McLaughlin reported the case of a horse used in a tallow-wagon which had a large swelling on the side of the neck, about the middle. Blistered; drove on one rein; dropped dead on the road. Also reported some mild cases of pneumonia and a case of fractured os coronæ.

Dr. Winchester reported an interesting case of ascites.

Dr. Cronan reported a horse with paralysis of the left side of the face, due to a blow, which has responded to treatment; also a case of scirrhous cord.

Dr. Winslow reported a case of a horse cutting his lip on a fence brushing flies.

Dr. Parker reported a case of the gradual recovery of a partial fracture of the cervical vertebræ, although there is still an enlargement; also reported an experience that he had with a herd of fifty cows, and pointed to the necessity of thorough disinfection and of removing all diseased animals from a herd.

Dr. Howard reported a case of lameness of a bay mare; lame behind. Diagnosed thrombus. Treatment—potassium iodide; complete recovery.

Dr. Stickney reported a case of a horse that ran into a barbed-wire fence, producing a wry-neck; for two years could not use its head, but finally recovered.

Meeting adjourned at 10.15 P.M. HENRY S. LEWIS,
Secretary.

